# Molecular and serological prevalence of *Toxoplasma gondii* and *Anaplasma* spp. infection in goats from Chongqing Municipality, China

**DOI:** 10.1051/parasite/2018024

**Published:** 2018-04-10

**Authors:** Zuoyong Zhou, Yutong Wu, Yiwang Chen, Zhiying Wang, Shijun Hu, Rongqiong Zhou, Chunxia Dong, Hongquan Lin, Kui Nie

**Affiliations:** 1 College of Animal Science, Rongchang Campus of Southwest University, No. 160 Xueyuan Road, Rongchang District, Chongqing 402460 PR China; 2 Veterinary Science Engineering Research Center of Chongqing, No. 160 Xueyuan Road, Rongchang District, Chongqing 402460 PR China; 3 Guizhou Institute of Animal Husbandry and Veterinary Medicine, No. 2 Laolipo, Naming District, Guizhou 550005 PR China; 4 Chongqing Animal Disease Prevention and Control Center, Chongqing 400174 PR China; 5 College of Animal Science and Technology, Southwest University, Beibei District Chongqing 400715 PR China

**Keywords:** *Toxoplasma gondii*, *Anaplasma* spp, Goat, Prevalence, Chongqing

## Abstract

Toxoplasmosis and anaplasmosis are severe zoonotic diseases, the former caused by *Toxoplasma gondii* and the latter by *Anaplasma* spp. In the present study, 332 goat blood samples were randomly collected from Chongqing Municipality, China to screen for *T. gondii* and *Anaplasma* spp. We used a polymerase chain reaction (PCR) to detect DNA, and enzyme-linked immunosorbent assay (ELISA) to test for *T. gondii* antibodies. The prevalence of *T. gondii* and *Anaplasma* spp. was 38% and 35% respectively by PCR, and 42% for *T. gondii* antibodies by ELISA. The co-infection rate by *T. gondii* and *Anaplasma* was 13%, where the two predominant pathogens co-infecting were *Anaplasma phagocytophilum* + *A. bovis* (10%), followed by *T. gondii* + *A. phagocytophilum* (9.64%). While co-infection by three pathogens varied ranging from 1.81% to 5.72%, less than 1% of goats were found to be positive for four pathogens. This is the first investigation of *T. gondii* and *Anaplasma* spp. infection in goats from Chongqing.

## Abstract (fr)

La toxoplasmose et l’anaplasmose sont des zoonoses sévères, la première causée par *Toxoplasma gondii* et la seconde par *Anaplasma* spp. Dans la présente étude, 332 échantillons de sang de chèvres ont été prélevés au hasard dans la municipalité de Chongqing en Chine pour détecter *T. gondii* et *Anaplasma* spp. L’ADN a été détecté par PCR, et les anticorps dirigés contre *T. gondii* par ELISA. La prévalence de *T. gondii* et *Anaplasma* spp. étaient respectivement de 38 % et 35 % par PCR, et de 42 % pour les anticorps anti-*T. gondii* par ELISA. Le taux de co-infection par *T. gondii* et *Anaplasma* était de 13 %, où la co-infection prédominante à deux pathogènes était *Anaplasma phagocytophilum* + *A. bovis* (10 %) suivie de *T. gondii* + *A. phagocytophilum* (9,64 %). Alors que la co-infection par trois agents pathogènes variait de 1,81 % à 5,72 %, moins de 1 % des chèvres ont été trouvés positives pour quatre pathogènes. Ceci est la première enquête sur les infections à *T. gondii* et *Anaplasma* spp. chez les chèvres de Chongqing.

## Introduction

Protozoan parasites and tick-borne infectious pathogens are common threats to both humans and animals [8,30]. The causative agent of toxoplasmosis, *Toxoplasma gondii*, is an obligate apicomplexan intracellular protozoan that can cause behavioral changes, neuropsychiatric disorders, abortions, stillbirth or fetal malformations, infertility and even death in humans and other mammals [19,21,24,26]. Anaplasmosis is caused by *Anaplasma*, a tick-borne pathogen that leads to inappetence, progressive anemia, fever, weight loss, milk production decrease, abortion, and sometimes death [14,18,25,34]. Infection by *T. gondii* and *Anaplasma* in goats not only affects the economic development of the animal industry, but can also have serious effects on human health [6,7,23]. Several surveys of *T. gondii* infection [20,31,32,33,40,41] or *Anaplasma* infection [18,36–38] in goats have been reported in some regions of China. However, they all focus only on *T. gondii* or *Anaplasma* infection; none examine co-infection by these pathogens. The presence of *A. phagocytophilum* can alter the immune system of the host and make the animal more susceptible to other parasitic agents [22]. It is important to study the relevance of this phenomenon regarding *T. gondii* and *Anaplasma* spp. infection in goats in Chongqing.

Chongqing Municipality is located in southwest China and has been incorporated into the national “Advantage of agricultural products regional planning”. It is recognized as a key area for goat breeding in China. However, there are no data on the prevalence of *T. gondii* and *Anaplasma* spp. infections in goats in Chongqing. The objective of this study was to investigate the prevalence of *T. gondii*, *Anaplasma* spp. and co-infection in goats in Chongqing, through detection of relevant pathogen DNA by PCR, and detection of *T. gondii* antibodies by enzyme-linked immunosorbent assay (ELISA).

## Materials and methods

### Collection of blood samples and DNA extraction

**Figure 1:**
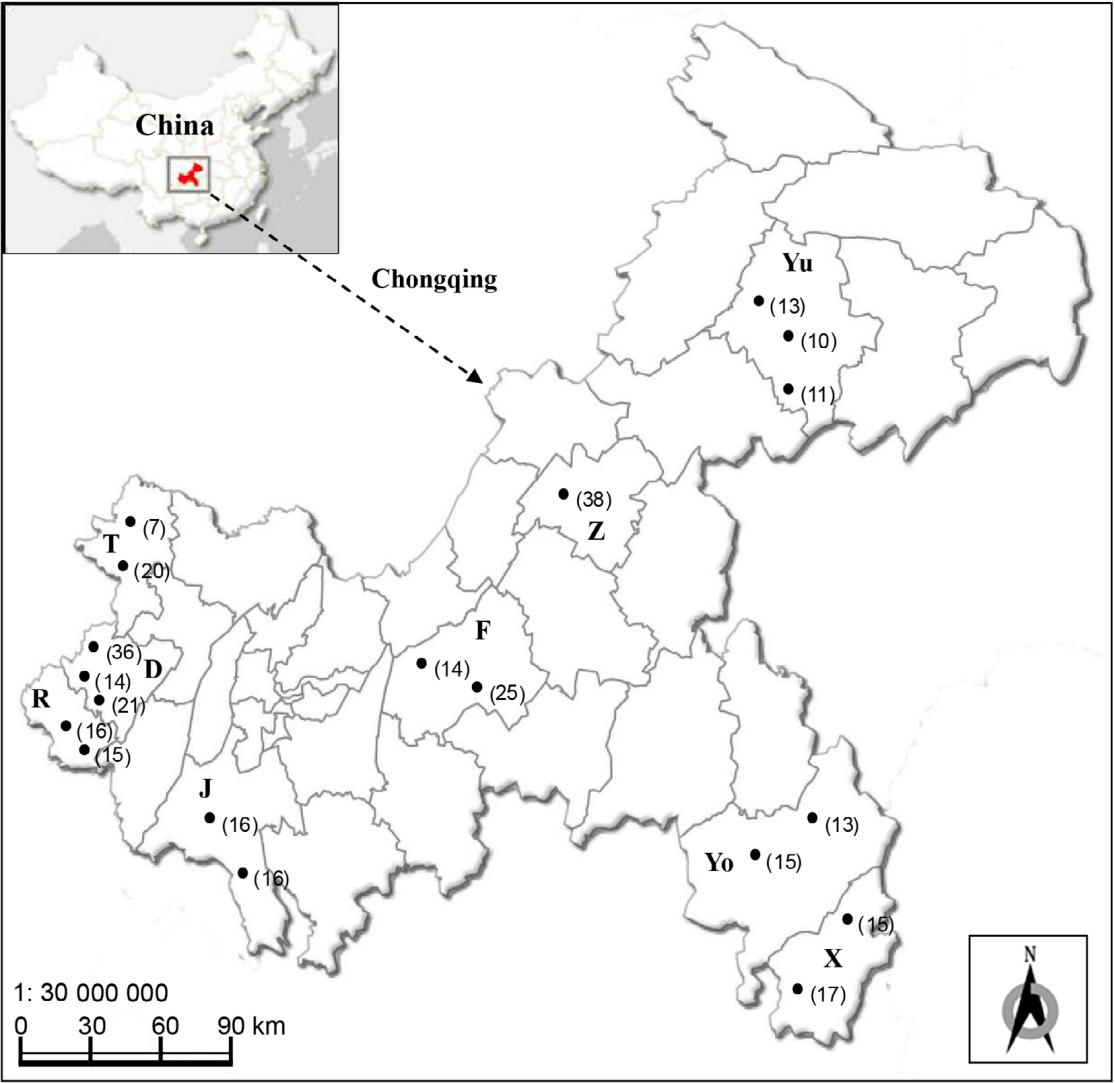
Map of surveyed counties located in Chongqing Municipality, China, where the blood samples of goats were collected. T: Tongnan; D: Dazu; R: Rongchang; J: Jiangjin; F: Fuling; Z: Zhongxian; Yu: Yunyang; Yo: Youyang; X: Xiushan. The black dots indicate the farms. The number of samples collected from the corresponding goat farm is indicated in parentheses.

The blood samples were collected from 332 apparently healthy goats randomly selected from 19 farms in 9 counties (Jiangjin, Dazu, Fuling, Rongchang, Tongnan, Youyang, Xiushan, Yunyang, and Zhongxian) of Chongqing (Fig. 1), from October 2014 to April 2016. The breeds of goats included the Boer goat, Dazu black goat, Chengdu ma goat, Nanjiang yellow goat, and Hybrid black goat. The sera were stored at −20 °C for *T. gondii* antibodies detection, and the blood samples were used for genomic DNA extraction using a Wizard^®^ Genomic DNA Purification Kit (Promega, Madison, WI, USA), according to the manufacturer’s instructions.

### Detection of *T. gondii* and *Anaplasma* DNA by PCR

Infections by *T. gondii* and *Anaplasma* spp. (*A. ovis*, *A. bovis*, and *A. phagocytophilum*) were detected by PCR in a reaction volume of 25 µL containing the following reagents: 12.5 µL of the PCR mix (2×) (Takara Dalian, China), 1 µL of each forward and reverse primer (100 µmol/L), 1 µL DNA (200 ng/µL) and 9.5 µL ddH_2_O. The amplified PCR products were separated by electrophoresis in 1.5% agarose gels. The primers and amplification conditions are listed in Table 1.

**Table 1:** Primers for *T. gondii* and *Anaplasma* detection in goats and PCR amplification conditions.

Pathogens	Methods	Primer	Amplicon (bp)	Thermocycler program				Cycles	Final extension	Reference
							
				Initial denaturation	Cycle					
*A. ovis*	PCR	5′-CCGGATCCTTAGCTGAACAGGAATCTTGC-3′ 5′-GGGAGCTCCTATGAATTACAGAGAATTGTTTAC-3′	867	94°C 30 s	94°C 30 s	60°C 30 s	58°C 1 min	30	72°C 5 min	[9]
*A. bovis*	PCR	5’-TCCTGGCTCAGAACGAACGCTGGCGGC-3’ 5’-AGTCACTGACCCAACCTTAAATGGCTG-3’	1433	94°C 5 min	94°C 30 s	55°C 30 s	72°C 30 s	30	72°C 10 min	[3]
	nPCR*	5′-CTCGTAGCTTGCTATGAGAAC-3′ 5′-TCTCCCGGACTCCAGTCTG-3′	551	94°C 5 min	94°C 30 s	55°C 30 s	72°C 30 s	30	72°C 10 min	[13]
*A. phagocytophilum*	PCR	5’-TCCTGGCTCAGAACGAACGCTGGCGGC-3’ 5’-AGTCACTGACCCAACCTTAAATGGCTG-3’	1433	94°C 5 min	94°C 30 s	55°C 30 s	72°C 30 s	30	72°C 10 min	[3]
	nPCR	5′-GCTGAATGTGGGGATAATTTAT-3′ 5′-ATGGCTGCTTCCTTTCGGTTA-3′	641	94°C 5 min	94°C 30 s	55°C 30 s	72°C 30 s	30	72°C 10 min	[13]
*T. gondii*	PCR	5’-CCGCGGAGCCGAAGTG −3’ 5’-TAGATCGCATTCCGGTGTCTC-3’	287	94°C 5 min	94°C 30 s	55°C 30 s	72°C 30 s	35	72°C 10 min	[17]
	nPCR	5’-GGACAGAAGTCGAAGGGGAC-3’ 5’-TTCCACCCTGCAGGAAAAGC −3’	181	94°C 5 min	94°C 30 s	55°C 30 s	72°C 30 s	30	72°C 5 min	[17]

* Nested PCR.

### Detection of *T. gondii* antibodies by ELISA

Serum antibodies against *T. gondii* were screened using the IDEXX Toxotest ELISA kit (IDEXX Laboratory, Westbrook, ME, USA), according to the manufacturer’s recommendations. The serum samples and controls were diluted to 1:400 and tested in duplicate. The optical density (OD) was measured at 450 nm with an ELISA plate reader (Thermo Fisher, Waltham, MA, USA). The S/P (samples/positive control) percent for each sample was calculated according to the formula: S/P% = (OD_450_ of the sample − OD_450_ of negative control)/(OD_450_ of positive control − OD_450_ of negative control) × 100. S/P% of samples less than 20 were considered negative for *T. gondii* antibodies. Samples with S/P% between 20 and 30 were considered questionable. If the S/P percentage was higher than or equal to 30, the samples were considered positive. If a sample remained suspect after a second run, a new sample from the same animal was collected and analyzed again. If the test result was again suspect, this sample was considered positive for *T. gondii* antibodies.

### Statistical analysis

The prevalence of *T. gondii* and *Anaplasma* infection in goats of different sexes and ages was analyzed using the Chi Square Test in SPSS (version 18.0, SPSS Inc., Chicago, IL, USA), and the probability (*p*) value of < 0.05 was considered statistically significant.

## Results and discussion

**Figure 2:**
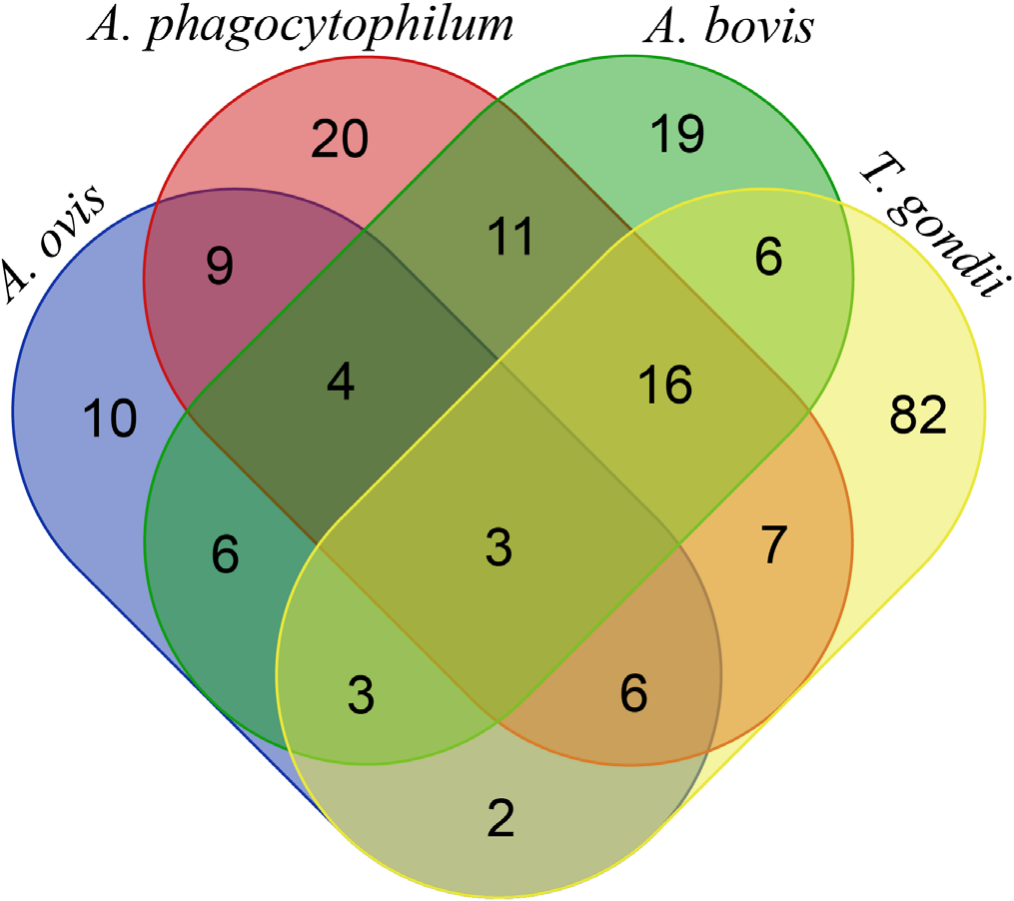
Venn diagram of mixed infection by *T. gondii* and *Anaplasma* in goats in Chongqing, southwest China. The number of goats tested positive for *T. gondii* and *Anaplasma* (*A. ovis*, *A. bovis* and *A. phagocytophilum*) infection is indicated by different colors in oval circles; the number of goats co-infected by pathogens is shown in the cross-over areas (n = 332).

The molecular prevalence of *T. gondii* was estimated to be 37.65% population, and the seroprevalence was 42.47% by ELISA (Table 2). The prevalence of *T. gondii* in goats has been reported to vary from 1.34% to 55.18% [1,2,4,11,12,29]. The relatively high prevalence of *T. gondii* in goats in Chongqing may be related to: 1) the oocysts of *T. gondii* excreted by infected cats that can easily develop to infective stages under the subtropical monsoon climate and humid weather in Chongqing and that are ingested by goats during grazing, and 2) the fact that most goats investigated in Chongqing were semi-housed, potentially increasing the risk of *T. gondii* sporulated oocyst ingestion in wild grazing conditions. The prevalence of *T. gondii* in goats in Chongqing Municipality was obviously higher than that of goats in other places in China, with the prevalence varying from 3.8% to 14.1% [16,20,31,32,40]. Similar to a previous report [31], the prevalence of *T. gondii* in female goats (39.91% by PCR and 44.96% by ELISA) in Chongqing was higher than that of males (31.91% by PCR and 36.17% by ELISA), and goats aged 1 year or more were more highly infected than those less than one year old. The overall prevalence of *Anaplasma* infection in goats in Chongqing was 34.94% (Table 2), which was comparable to *Anaplasma* infection in Yunnan and Henan provinces (36.5%) [39], but higher than rates reported by other investigators for goats [36,37] and lower than rates for goats from Henan, Guizhou, Zhejiang and Hubei provinces in China [18]. Contrary to the prevalence of *T. gondii* in goats by sex, the prevalence of *Anaplasma* (37.23%) was higher in males than in females (34.03%). The prevalence of *Anaplasma* in goats aged one year or more (37.96%) was higher than that in goats less than 1 year old (29.31%). This is consistent with other reports [5,15]. The difference could be due to the fact that older animals are exposed to several tick seasons [5] and have a greater chance of exposure to ticks carrying *Anaplasma* spp. [15]. Similar to a previous study [37], 22.89% (76/332) of goats were positive for *A. phagocytophilum* infection followed by *A. bovis* (62/332, 18.67%) and *A. ovis* (43/332, 12.95%), which was not consistent with other reports indicating that the prevalence of *A. phagocytophilum* in goats was lower than that of *A. bovis* and *A. ovis* [18]. In addition, the prevalence of *A. phagocytophilum* in goats in this study was higher than that of goats in Slovakia [8]. Unlike a previous report [18], the dominant co-infection of *A. phagocytophilum* + *A. bovis* (34/332, 10.24%) was higher than *A. phagocytophilum* + *A. ovis* (22/332, 6.63%) and *A. bovis* *+* *A. ovis* (16/332, 4.82%). Co-infection by three *Anaplasma* spp. occurred in only 2.11% of the goats studied, which is similar to the other report [18] (Fig. 2).

**Table 2:** Overall prevalence of *T. gondii* and *Anaplasma* infection in goats in Chongqing, southwest China tested by PCR and ELISA.

Variables	Prevalence of *T. gondii* infection (%)					Prevalence of *Anaplasma* infection (%)	
			
	Prevalence by PCR (positive/examined)	95% CI*	Prevalence by ELISA (positive/examined)	95% CI		Prevalence by PCR (positive/examined)	95% CI
Location							
Jiangjin	34.38 (11/32)	18.57-53.19	31.25 (10/32)	16.12-50.01		0 (0/32)	0.00-10.89
Dazu	50.70 (36/71)	38.56-62.78	60.56 (43/71)	48.25-71.97		14.08 (10/71)	6.97-24.38
Fuling	33.33 (13/39)	19.09-50.22	41.03 (16/39)	25.57-57.90		20.51 (8/39)	9.30-36.46
Rongchang	45.16 (14/31)	27.32-63.97	35.48 (11/31)	19.23-54.63		38.71 (12/31)	21.85-57.81
Tongnan	29.63 (8/27)	13.75-50.18	44.44 (12/27)	25.48-64.67		77.78 (21/27)	57.74-91.38
Youyang	35.71 (10/28)	18.64-55.93	39.29 (11/28)	21.50-59.42		78.57 (22/28)	59.05-91.70
Xiushan	37.50 (12/32)	21.10-56.31	53.13 (17/32)	34.74-70.91		56.25 (18/32)	37.66-73.64
Yunyang	41.18 (14/34)	24.65-59.30	41.18 (14/34)	24.65-59.30		41.18 (14/34)	24.65-59.30
Zhongxian	18.42 (7/38)	7.74-34.33	18.42 (7/38)	7.74-34.33		28.95 (11/38)	15.42-45.90
							
Gender							
Male	31.91 (30/94)	22.67-42.33	36.17 (34/94)	26.51-46.73		37.23 (35/94)	27.48-47.82
Female	39.92 (95/238)	33.64-46.44	44.96 (107/238)	38.53-51.52		34.03 (81/238)	28.04-40.43
							
Age							
< 1 year	37.07 (43/116)	28.29-46.53	38.79 (45/116)	29.89-48.28		29.31 (34/116)	21.23-38.48
≥1 year	37.96 (82/216)	31.47-44.80	44.44 (96/216)	37.70-51.34		37.96 (82/216)	31.47-44.80
Total	37.65 (125/332)	32.42-43.10	42.47 (141/332)	37.09-47.98		34.94 (116/332)	29.82-40.34

* 95% confidence intervals.

Co-infection by *T. gondii* and *Anaplasma* has been reported in rodents [27], dogs [10], ticks [35], and wild boars [22]. However, a survey on the occurrence of goats co-infected by *T. gondii* and *Anaplasma* was only reported in Slovakia [8]. In this study, 43 out of 332 (12.95%) goats were positive for *T. gondii* and *Anaplasma* (Fig. 2), indicating a relatively high prevalence of these two pathogens. The dominant co-infection between *T. gondii* and a single *Anaplasma* species was *T. gondii* + *A. phagocytophilum* (9.64%, 32/332), followed by *T. gondii* *+* *A. bovis* (8.43%, 28/332) and *T. gondii* + *A. ovis* (4.22%, 14/332). The high prevalence of *A. phagocytophilum* and *T. gondii* co-infection confirms the hypothesis that the presence of *A. phagocytophilum* can alter the immune system of the host and make the animal more susceptible to other parasitic agents [22]. *A. bovis* was first reported to infect goats in China by Liu *et al.* (2012). The high prevalence of *A. bovis* in this study also confirmed that goats may be an important natural reservoir for this organism [18]. Three-pathogen co-infection by *A. ovis* + *A. phagocytophilum* + *T. gondii*, *A. phagocytophilum* *+* *A. bovis* + *T. gondii*, and *A. bovis* + *A. ovis* + *T. gondii* was 2.71% (9/332), 5.72% (19/332) and 1.81% (6/332) respectively. Four-pathogen co-infection (*A. ovis*, *A. bovis*, *A. phagocytophilum* and *T. gondii*) was simultaneously detected in 3 goats (0.9%) (Fig. 2). The main species of tick in Chongqing is *Boophilus microplus* [28], which is one of the vectors of *Anaplasma phagocytophilum* in China [37]. Since *T. gondii* has already been detected in ticks [35], a study of *T. gondii* and *Anaplasma* carriage by *Boophilus microplus* in Chongqing should be carried out.

## Conflict of interest

None of the authors have any conflict of interest.
